# Recurrent novel *HMGA2-NCOR2* fusions characterize a subset of keratin-positive giant cell-rich soft tissue tumors

**DOI:** 10.1038/s41379-021-00789-8

**Published:** 2021-03-19

**Authors:** Abbas Agaimy, Michael Michal, Robert Stoehr, Fulvia Ferrazzi, Pavel Fabian, Michal Michal, Alessandro Franchi, Florian Haller, Andrew L. Folpe, Kemal Kösemehmetoğlu

**Affiliations:** 1 Institute of Pathology, Friedrich Alexander University Erlangen-Nürnberg, University Hospital of Erlangen, Erlangen, Germany; 2 Department of Pathology, Charles University, Faculty of Medicine in Plzen, Plzen, Czech Republic; 3 Bioptical Laboratory, Ltd, Plzen, Czech Republic; 4 Department of Nephropathology, Institute of Pathology, Friedrich-Alexander-University Erlangen-Nürnberg, Erlangen, Germany; 5 Department of Oncological and Experimental Pathology, Cancer Institute, Brno, Czech Republic; 6 Department of Translational Research, University of Pisa, School of Medicine, Pisa, Italy; 7 Department of Laboratory Medicine and Pathology, Mayo Clinic, Rochester, MN USA; 8 Department of Pathology, Faculty of Medicine, Hacettepe University, Ankara, Turkey

**Keywords:** Pathogenesis, Diseases

## Abstract

Giant cell tumors of soft tissue (GCT-ST) are rare low-grade neoplasms that were at one time thought to represent the soft tissue counterparts of GCT of bone (GCT-B) but are now known to lack the *H3F3* mutations characteristic of osseous GCT. We present six distinctive giant cell-rich soft tissue neoplasms that expressed keratins and carried a recurrent *HMGA2-NCOR2* gene fusion. Patients were five females and one male aged 14–60 years (median, 29). All presented with superficial (subcutaneous) masses that were removed by conservative marginal (3) or wide (2) local excision. The tumors originated in the upper extremity (2), lower extremity (2), head/neck (1), and trunk (1). Five patients with follow-up (median, 21 months; range, 14–168) remained disease-free. Grossly, all tumors were well-demarcated but not encapsulated with variable lobulation. Histologically, they were composed of bland plump epithelioid or ovoid to spindled mononuclear cells admixed with evenly distributed multinucleated osteoclast-type giant cells. Foci of stromal hemorrhage and hemosiderin were seen in all cases. The mitotic activity ranged from 2 to 14/10 high power fields (median: 10). Foci of necrosis and vascular invasion were seen in one case each. The mononuclear cells were immunoreactive with the AE1/AE3 keratin cocktail and less frequently/less diffusely for K7 and K19 but lacked expression of other lineage-associated markers. RNA-based next-generation sequencing revealed an *HMGA2-NCOR2* fusion in all tumors. None of the keratin-negative conventional GCT-ST showed the *HMGA2-NCOR2* fusion (0/7). Metaplastic bone (4/9) and SATB2 expression (3/4) were frequent in keratin-negative conventional GCT-ST but were lacking in keratin-positive *HMGA2-NCOR2* fusion-positive tumors. The distinctive immunophenotype and genotype of these tumors strongly suggest that they represent a discrete entity, differing from conventional GCT-ST and other osteoclast-rich morphologic mimics. Their natural history appears favorable, although a study of additional cases and longer follow-up are warranted.

## Introduction

The presence of an evenly distributed osteoclastic giant cell component is a well-known phenomenon in soft tissue and bone neoplasms. This readily recognizable morphological feature occurs in two different settings: (1) neoplasms that are definitionally giant cell-rich and are hence named after this feature such as giant cell tumor of bone (GCT-B) [1] and giant cell tumor of soft tissue (GCT-ST) [2], and (2) neoplasms that only occasionally or rarely display a prominent evenly distributed giant cell component. The latter include subsets of osteosarcoma [3], leiomyosarcoma [4], epithelioid sarcoma [5], and others. Accordingly, the exact subtyping of any giant cell-rich lesion is based on the identification and precise phenotyping of the neoplastic mononuclear component. In routine practice, subtyping is achieved via a set of defined phenotypic (such as demonstration of myoid markers in giant cell-rich leiomyosarcoma) [4], genotypic (e.g., presence of *H3F3* mutations by molecular testing or using the mutation-specific H3.3 G34W antibody in GCT-B) [6–10] or both features (e.g. demonstration of epithelial phenotype and *SMARCB1* inactivation in epithelioid sarcoma) [5].

Bland giant cell-rich soft tissue lesions are heterogeneous. They encompass extra-osseous aneurysmal cysts of soft tissue [11], a subset of giant cell-rich nodular fasciitis, plexiform fibrohistiocytic tumors [12, 13], and a group of lesions histologically indistinguishable from GCT-B, for which different terminologies have evolved [14–18]. “Giant cell tumor of soft tissue” is the currently accepted WHO terminology for the latter [2].

The molecular pathogenesis of GCT-ST remained obscure as they lack the *H3F3* mutations seen in their intraosseous counterparts [19, 20]. We herein describe clinicopathological and molecular features of a distinctive molecular subtype of GCT-ST characterized by keratin immunoreactivity and recurrent gene fusion involving *HMGA2* and *NCOR2*.

## Materials and methods

We recently encountered a keratin-positive GCT-ST (Case 1 in Table 1), which we submitted to molecular analyses according to our institution policy of sending soft tissue tumors with unknown genetics for molecular investigation. We detected an *HMGA2-NCOR2* fusion in this tumor. Notably, we have not encountered this fusion in >800 other soft tissue neoplasms tested with the same RNA Panel over the last 4 years. To test the hypothesis that this fusion is potentially specific to this rare soft tissue lesion, we retrieved 14 additional tumors diagnosed as GCT-ST or other giant cell-rich soft tissue lesions from our consultation files to test them for keratin expression and presence of the same or other gene fusions using the RNA fusion panel. Immunohistochemistry (IHC) was performed on 3-µm sections cut from paraffin blocks using a fully automated system (“Benchmark XT System”, Ventana Medical Systems Inc., 1910 Innovation Park Drive, Tucson, Arizona, USA) and the following antibodies: keratin cocktail (clones AE1/AE3, 1:40, Zytomed, Berlin, Germany), K5 (clone XM26, 1: 50, Zytomed), K7 (OV-TL, 1:1000, Biogenex), K19 (RCK108, 1:300, Dako), low-molecular weight keratins (clone CAM5.2, ready-to-use, CellMarque), pankeratin (clone OSCAR, ready-to-use, CellMarque), CD68 (clone PGM1, 1:200, Dako), CD163 (clone 10D6, 1:500, Novocastra), p63 (SFI-6, 1:100, DCS), desmin (clone D33, 1:250, Dako), alpha smooth muscle actin (clone 1A4, 1:200, Dako), HMB45 (clone HMB45, 1:50, Enzo), ERG (EPR3864, prediluted, Ventana), S100 protein (polyclonal, 1:2500, Dako), SATB2 (clone EPNCIR130A, 1:200, Abcam) and SMARCB1/INI1 (clone MRQ-27, dilution, 1:50, Zytomed). The H3.3 G34W IHC was performed manually using a mutation-specific antibody (clone RM 263, 1:500, BD Biosciences). Tissue microarray slides containing multiple GCT-B were used as an external control for the H3.3 G34W antibody. Only “clean” nuclear H3.3 G34W antibody staining without background staining was considered positive. Samples were used in accordance with ethical guidelines for the use of retrospective tissue samples provided by the local ethics committee of the Friedrich-Alexander University Erlangen-Nuremberg (ethics committee statements 24.01.2005 and 18.01.2012).

**Table 1:** Clinicopathological features of keratin-positive (1–6) and keratin-negative (7–15) giant cell-rich tumors of soft tissue.

	Age/sex	Site	Size (cm)	Depth	Treatment	Outcome
1	25/F	Right upper arm	2 cm in aggregate	Subcutaneous	Excision—marginal	NED (6 mo)
2	26/F	Right knee medial	2.8 cm	Subcutaneous	Wide excision (R0) + CT (4 courses Adriamycin 85 mg infusion)	NED (12 mo)
3	60/F	Right forearm	NA	Skin/subcutaneous	Excision (R0)	NED (14 years)
4	33/F	Right flank	2.5 cm in aggregate	Subcutaneous	Excision, fragmented (Rx)	NED (31 mo)
5	14/M	Angle of jaw (right)	3 cm	Subcutaneous	Excision—marginal	NED (21 mo)
6	50/F	Shin	2 cm	Subcutaneous	Biopsy	NA
7	78/M	Neck	2 cm	Subcutaneous	Excision	NED (4 mo)
8	57/M	Hand	2.2 cm	Subcutaneous	Excision	NED (20 mo)
9	20/M	Arm	4.5 cm	Subcutaneous	Excision	Local recurrence (101 mo)
10	11/M	Leg	1.5 cm	Subcutaneous	Excision	Recent case
11	34/F	Arm	NA	Skin	Biopsy	NA
12	48/F	Forearm	NA	Subcutaneous	Biopsy	NA
13	75/F	Elbow	NA	Skin/subcutaneous	Biopsy	NA
14	72/M	Finger	NA	Skin/subcutaneous	Excision	NA
15	25/F	Dorsal foot	NA	Skin	Excision	NA

*CT* chemotherapy, *F* female, *M* male, *mo* month, *NA* not available, *NED* no evidence of disease.

Statistical analysis for the comparison of keratin-positive and keratin-negative giant cell-rich soft tissue tumor subcohorts was performed using JMP SAS V.15.1.0 (SAS). Variables did not show normal distribution, thus the nonparametric Mann–Whitney *U* test was used for continuous variables. Grouped variables were compared using Fisher’s exact tests.

### Next-generation sequencing

RNA was isolated from formalin-fixed paraffin-embedded (FFPE) tissue sections using RNeasy FFPE Kit of Qiagen (Hilden, Germany) and quantified spectrophotometrically using NanoDrop-1000 (Waltham, United States). Molecular analysis was performed using the TruSight RNA Fusion Panel (Illumina, Inc., San Diego, CA, USA) with 500 ng RNA as input according to the manufacturer’s protocol. Libraries were sequenced on a MiSeq (Illumina, Inc., San Diego, CA, USA) with >3 million reads per case, and sequences were analyzed using the RNA-Seq Alignment workflow, version 2.0.1 (Illumina, Inc., San Diego, CA, USA). The RNA-Seq alignment app (Illumina) was employed to call fusions by using the TopHat-Fusion algorithm, and to generate raw counts for each of the targeted 507 genes. Additionally, the Integrative Genomics Viewer (IGV), version 2.2.13 (Broad Institute, REF) was used for data visualization of fusions. For samples 6, 8, and 11, the fusion calling algorithm revealed a failure of quality control parameters, and no fusion was called. However, visual inspection of the automatically generated bam files revealed an *HMGA2*-*NCOR2* gene fusion in sample 6 at low coverage, but no fusion in samples 8 and 11.

### Differential expression and gene set enrichment analyses

Samples 6, 8, and 11 were excluded from gene expression analysis as they had total raw read counts <50,000 compared to read counts >1,000,000 in all other cases. Differential expression analysis was performed within the R/Bioconductor environment v.4.0.3 [21, 22] relying on the DESeq2 package v.1.30.0 [23]. Genes with an adjusted *p* value (Benjamini–Hochberg correction) < 0.01 were considered differentially expressed. Principal component analysis was based on the variance-stabilized transformed counts of the 100 genes with the highest variance across all samples. The heatmap was done using the variance-stabilized transformed count matrix of the differentially expressed genes as input relying on the gplots package v.3.1.0 [24]. Functional enrichment analyses of the differentially expressed genes were performed with DAVID database [25], using the 507 gene list of the panel as background. Although the TruSight RNA fusion panel was not developed for the analysis of differential gene expression, we have recently employed this method successfully in comparing different subgroups of solitary fibrous tumors [26], while a larger version of this panel has been used to classify hematological neoplasms by gene expression [27].

## Results

### General features of the study cohort

The clinicopathological features of the study cohort are summarized in Table 1. Of 17 initially retrieved tumors, two were excluded upon critical reevaluation. One tumor (1.5 cm) originating in the lower arm of a 10-year-old male represented a giant cell-rich tenosynovial giant cell tumor. Another 12 cm tumor in the left calf and fibula of a 30-year-old male represented a keratin-positive high-grade epithelioid malignancy with a few scattered giant cells and was excluded as well. The remaining 15 cases were then split into a keratin-positive (*n* = 6) and a keratin-negative (*n* = 9) subcohorts irrespective of their other histological, immunohistochemical, or molecular features.

### Keratin-positive giant cell-rich soft tissue tumors

**Fig. 1:**
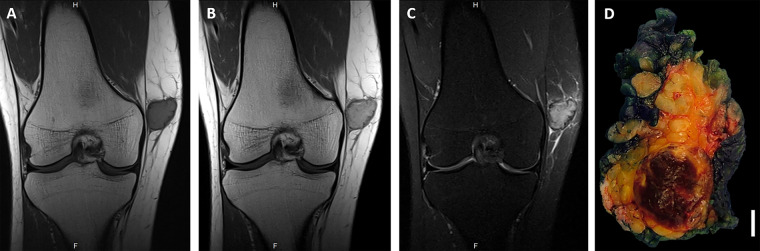
Representative images of the radiological (**A**–**C**) and gross (**D**) features of *HMGA2-NCOR2* fusion-positive giant cell tumors (Case 2). Coronal T1-weighted (T1W) pre- (**A**) and post-contrast (**B**) and proton-density (**C**) MR images show an enhancing deep subcutaneous mass that abuts the peripheral fascia at the distal medial aspect of the right thigh. The slight hyperintensity of the tumor relative to muscle on pre-contrast T1W images and the peripheral low signal on all sequences likely represent its hemorrhagic content. Mild reticular edema surrounds the lesion. The epicenter of the tumor is in the subcutaneous tissue with well-circumscribed borders and tan to brown lobulated cut-surface (**D**).

Six cases were diffusely positive with the AE1/AE3 keratin cocktail. These six tumors affected five females and one male aged 14–60 years (median: 29 years). All presented with superficial (subcutaneous) masses that underwent conservative marginal (3) or wide (2) local excision. One case was biopsied only. Two tumors originated in the upper extremity, two in the lower extremity and one each in the head and neck, and the trunk. Their size range was 2–3 cm (median, 2.5). Follow-up was available for five cases and ranged from 6 to 168 months (median, 21); no recurrences or distant metastases have been recorded during this follow-up period. Case 2 exhibited a vascular invasion and the patient presented with an enlarged (1 cm in diameter) PET-positive lymph node in the right inguinal area. She also had a 1 cm nodule at the right arm which was histologically diagnosed (post-chemotherapy) as organizing thrombus. Both findings disappeared after chemotherapy and might have represented tumor manifestations that healed after chemotherapy. None of the patients had evidence of another keratin-positive malignancy. Representative radiological images of one case are depicted in Fig. 1A–C.

### Pathological findings

The histopathological and immunohistochemical features of the cases are summarized in Table 2. Grossly, all tumors were well-circumscribed, but unencapsulated. The cut-surface was described as tan to brown with vague lobulation (Fig. 1D).

**Table 2:** Pathological, immunohistochemical, and molecular findings in keratin-positive (1–6) and keratin-negative (7–15) giant cell-rich tumors of soft tissue.

	Lobulated/multinodular	Mitoses/10 hpfs	Necrosis	Vascular invasion	Stromal hemorrhage	Metaplastic bone	Aneurysmal features	Peritumoral lymphoid reaction	CK IHC	SATB2	TruSight RNA fusion panel
1	Uninodular lobulated	3	Absent	Absent	Present	Absent	Absent	Present	AE1/AE3+++ CK7+, CK19+	−	HMGA2-NCOR2 Ex 3/Ex 16 chr12:66232348:+ chr12:124887107:−
2	Uninodular lobulated	2	Absent	Present	Present	Absent	Absent	Present	AE1/AE3+++, Cam5.2+	−	HMGA2-NCOR2 Ex 3/Ex 20 chr12:66232348:+ chr12:124862929:−
3	Uninodular vaguely lobular	14	Present	Absent	Present	Absent	Absent	Present	AE1/AE3+++, OSCAR+++, CK7 (F+), CK34Beta12-, CK19−	−	HMGA2-NCOR2 Ex 3/Ex 16 chr12:66232348:+ chr12:124887107:−
4	Uninodular vaguely lobular	13	Absent	Absent	Present	Absent	Absent	Absent	AE1/AE3+++, OSCAR++, CK7 (F+), CK19 (i+)	−	HMGA2-NCOR2 Ex 3/Ex 16 chr12:66232348:+ chr12:124887107:−
5	Uninodular lobulated	10	Absent	Absent	Present	Absent	Focal	Present	AE1/AE3++, CK19 (i+) CK7−	−	HMGA2-NCOR2 Ex 3/Ex 16 chr12:66232348:+ chr12:124887107:−
6	Multinodular lobulated	1	Absent	Absent	Present	Absent	Focal	Absent	AE1/AE3+++	ND	HMGA2-NCOR2 Ex 3/Ex 20 chr12:66232348:+ chr12:124862929:−
7	Uninodular lobulated encapsulated	2	Present	Absent	Present	Present	Absent	Absent	AE1/AE3−	+	No fusion
8	Multinodular lobulated	17	Absent	Absent	Present	Present	Present	Absent	AE1/AE3−	+	Failed
9	Uninodular	4	Absent	Absent	Present	Present	Present	Absent	AE1/AE3−	+	No fusion
10	Poorly marginated unilobular	5	Absent	Absent	Present	Absent	Absent	Absent	AE1/AE3−	−	No fusion
11	Multinodular lobulated	1	Absent	Absent	Present	Absent	Absent	Absent	AE1/AE3−	ND	Failed
12	Uninodular lobulated	3	Present	Absent	Present	Absent	Absent	Absent	AE1/AE3−	ND	No fusion
13	Plexiform	0	Absent	Absent	Present	Absent	Absent	Absent	AE1/AE3−	ND	No fusion
14	Uninodular lobulated encapsulated	3	Absent	Absent	Present	Present	Absent	Absent	AE1/AE3−	ND	No fusion
15	Circumscribed not lobulated	2	Absent	Absent	Present	Absent	Absent	Absent	AE1/AE3−	ND	ALK-PPFIBP1 IHC: D5F3+)

*CK* cytokeratin; *F* focal, *i* isolated cells, *IHC* immunohistochemistry, *ND* not done.

**Fig. 2:**
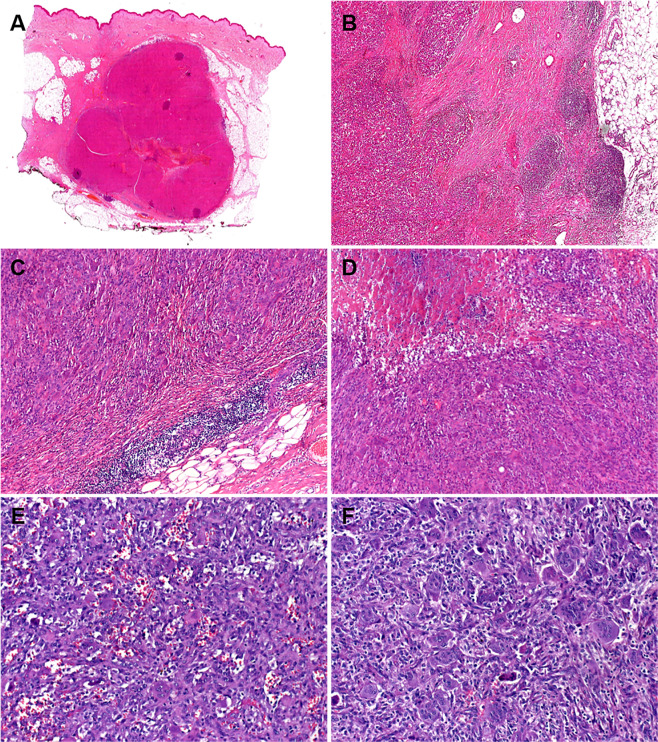
Representative histological images of *HMGA2-NCOR2* fusion-positive giant cell tumors. At low-power, the subcutaneous tumor is well circumscribed with vague lobulation and a central zone of ischemic-type necrosis (**A**). Variable lobulation is seen (**B**). Pericapsular lymphoid aggregates are consistent findings (**C**). **D** Higher magnification of the ischemic-type necrosis. **E** Cellular areas with pseudoangiomatous or aneurysmal stromal changes. **F** Numerous multinucleated osteoclast-type giant cells are evenly distributed among the mononuclear bland cell component.

**Fig. 3:**
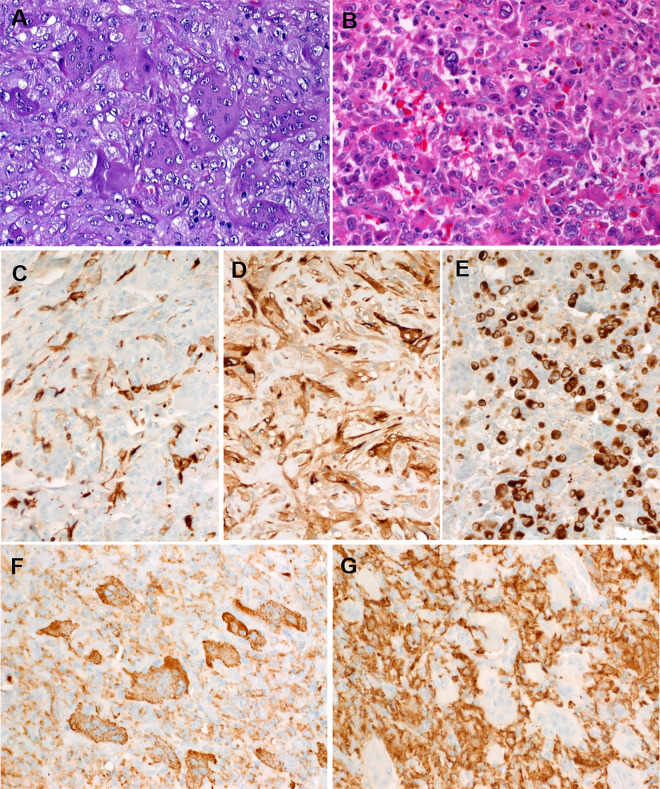
Representative histological images of *HMGA2-NCOR2* fusion-positive giant cell tumors. **A** Higher magnification of the two cell populations. **B** The neoplastic mononuclear cell component of this tumor (Case 2) had predominantly small epithelioid or plasmacytoid morphology. The AE1/AE3 (**C**) and OSCAR (**D**) keratin cocktails highlighted the mononuclear cell component with a variable dendritic pattern. **E** The case shown in **B** revealed a prominent paranuclear keratin staining pattern (AE1/AE3) consistent with the plasmacytoid cell morphology. **F** CD68 was strongly positive in both cell populations highlighting the giant cell component. **G** On the other hand, CD163 was expressed only in the mononuclear cell component.

At low power, the tumors were all uninodular, but showed variable lobulation and were occasionally arranged into plexiform lobules of variable size. These lobules were bordered by fibrosclerotic hyaline connective tissue bands (Fig. 2A, B). A variably intense lymphoid reaction at the periphery of the tumor was seen in all cases (Fig. 2C). Foci of ischemic-type necrosis were seen (Fig. 2D). Notably, metaplastic bone or a peripheral shell of mature bone was uniformly absent (Fig. 2E, F). The tumors were predominantly centered within the subcutaneous tissue (Fig. 2A) and were composed of plump epithelioid or ovoid mononuclear cells with vesicular chromatin, variably prominent nucleoli, and a rim of pale eosinophilic cytoplasm with indistinct cell borders (Fig. 3A). Occasional subtle areas with plasmacytoid cytology were observed but only after a careful search. One tumor however showed predominant epithelioid or plasmacytoid morphology (Fig. 3B). The mononuclear neoplastic cells formed diffuse solid sheets interrupted by a prominent component of evenly distributed multinucleated giant cells of osteoclast type. The number of nuclei within the giant cells varied greatly from >10 (Fig. 3A) to few (Fig. 3B). A variable degree of stromal hemorrhage and hemosiderin deposition was seen in all cases. The mitotic activity ranged from 2 to 14 mitoses per 10 high power fields (median: 10). Foci of necrosis and vascular invasion were seen in one case each.

### Immunohistochemical features

By IHC, all tumors were positive for the AE1/AE3 keratin cocktail in the mononuclear cell population with a diffuse cytoplasmic pattern, occasionally highlighting dendritic-like cytoplasmic extensions (Fig. 3C). Other keratin markers (OSCAR, Cam5.2) were variably positive (Fig. 3D). The one case with plasmacytoid morphology (Case 2) showed distinctive paranuclear reactivity with AE1/E3 (Fig. 3E). K7 was positive in two of three cases. Three of four tumors showed prominent (one case) or single cell (two cases) K19 immunoreactivity. S100 protein was tested in four cases with one case showing limited focal reactivity. The giant cell component was highlighted by CD68 (Fig. 3F) but was negative for CD163 (Fig. 3G). The mononuclear cells revealed variable diffuse reactivity with CD68 (Fig. 3F) and CD163 with more intense staining seen with CD163 (Fig. 3G). SATB2 was negative in all cases (0/5). SMARCB1/INI1 showed retained nuclear reactivity. All six tumors lacked nuclear reactivity with the mutation-specific H3.3 G34W antibody. All other lineage-specific markers including endothelial, melanocytic, myogenic, myoepithelial, and neurogenic (done initially on a single case-based approach) were negative.

### Molecular findings

**Fig. 4:**
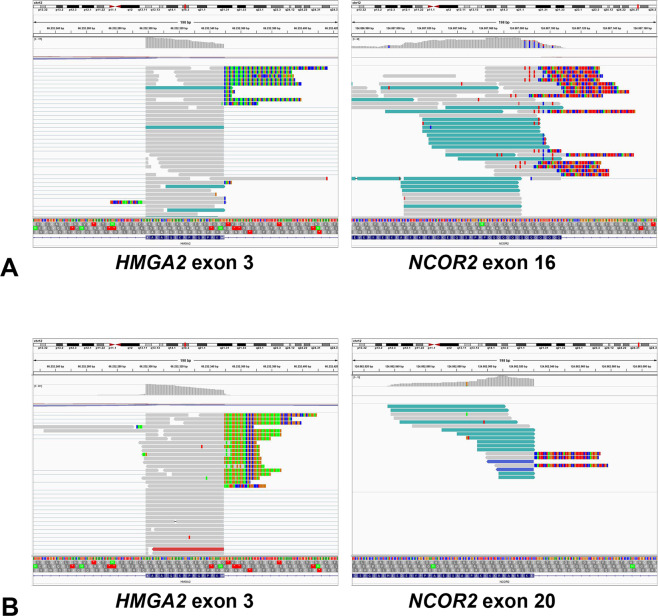
Representative integrative genomics viewer (IGV) screenshots of two cases with *HMGA2*-*NCOR2* gene fusion. **A** Case 1 with *HMGA2* exon 3—*NCOR2* exon 16 gene fusion. Note both split reads and paired reads at the 3′-border of *HMGA2* exon 3 (left) corresponding to the 5′-end of *NCOR2* exon 16 (right). **B** Case 2 with *HMGA2* exon 3—*NCOR2* exon 20 gene fusion. Note both split reads and paired reads at the 3′-border of *HMGA2* exon 3 (left) corresponding to the 5′-end of *NCOR2* exon 20 (right).

All six keratin-positive tumors revealed an *HMGA2-NCOR2* fusion (Table 2), with the 3′- end of *HMGA2* exon 3 involved in all six cases. Regarding the *NCOR2* fusion partner, four tumors had the breakpoint at the 5′-end of exon 16, and two cases had the breakpoint at the 5′-end of exon 20 (Fig. 4). Regarding the putative chimeric HMGA2-NCOR2 fusion protein, a DNA-binding motif encoded by *HMGA2* exons 1–3 was included in the N-terminal part, while two repressor domains, a binding site for histone deacetylase 3 (HDAC3) as well as binding sites for multiple nuclear hormone receptors and transcription factors were included in the C-terminal part encoded by *NCOR2* exons 16–47. A SANT/MYB domain encoded by *NCOR2* exons 11–17 was partially included in the *HMGA2-NCOR2* fusion variant with a breakpoint at *NCOR2* exon 16, but not in the two cases with breakpoints at *NCOR2* exon 20.

**Fig. 5:**
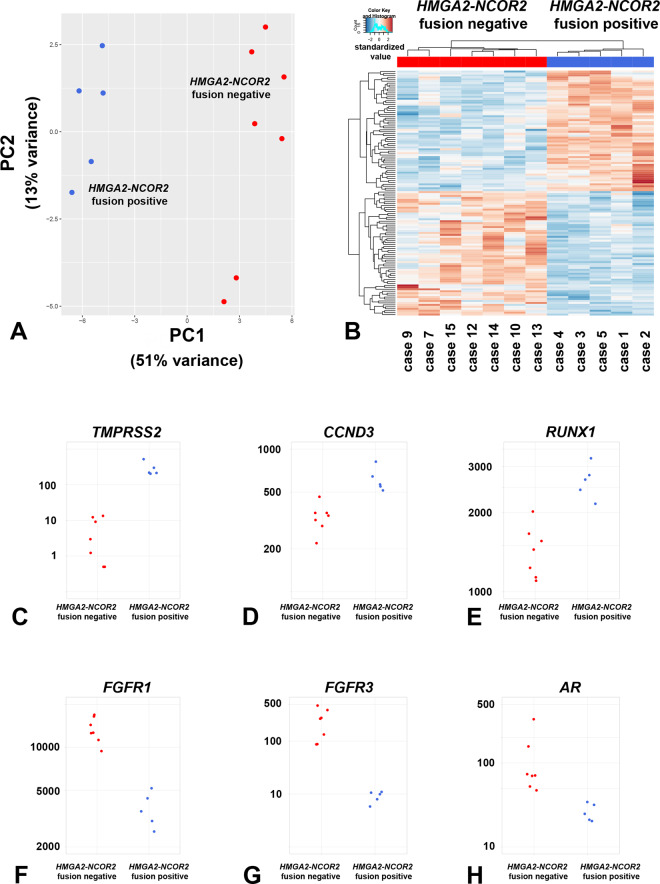
Visualization of differential gene expression comparing tumors with and without the *HMGA2*-*NCOR2* gene fusion. **A** Principal component analysis based on the normalized gene expression levels of 100 genes from the 507 gene panel with the highest variability of expression among the whole cohort. Note that the tumors show distinct clustering correlating to the presence (blue dots) or absence (red dots) of the *HMGA2*-*NCOR2* gene fusion. Each dot represents a single case. **B** Cluster analysis and heatmap visualization of five tumors with *HMGA2*-*NCOA2* gene fusion (blue) compared to seven tumors without *HMGA2*-*NCOA2* gene fusion (red), based on the expression of 126 significantly differentially expressed genes. Note the homogenous gene expression pattern between both groups. Each row represents a gene and each column represents a tumor, with the normalized gene expression level indicated by color code. **C**–**E** Normalized read counts for the genes *TMPRSS2*, *CCND3*, and *RUNX1* that were significantly higher expressed in the *HMGA*-*NCOR2* fusion-positive tumors. **F**–**H** Normalized read counts for the genes *FGFR1*, *FGFR3*, and *AR* that were significantly higher expressed in the *HMGA*-*NCOR2* fusion negative tumors. Each dot represents a single tumor.

The RNA fusion panel also allows the quantification expression profiles associated with the tumors, as previously shown by us and others [26, 27]. An unsupervised principal component analysis based on the expression of the 100 genes with the highest variability across the whole cohort revealed that tumors with and without the *HMGA2*-*NCOR2* fusion clustered in two distinct groups (Fig. 5A). Differential expression analysis of the 507 genes comprised in the RNA fusion panel showed that, 64 genes had a significantly higher expression in the group of keratin-positive tumors with *HMGA2*-*NCOR2* fusion, while 62 genes were significantly higher expressed in the group of keratin-negative tumors lacking an *HMGA2*-*NCOR2* fusion (Supplementary Table 1, Fig. 5B). Genes that were higher expressed in the tumors with *HMGA2*-*NCOR2* fusion included *TMPRSS2*, *CCND3*, and *RUNX1*, as well as several genes involved in T-cell signaling (e.g. *TCL1A*, *IRF4*, and *POU2AF1* among the top five higher expressed genes) (Fig. 5C–E). In contrast, *FGFR1*, *FGFR3*, and *AR* encoding for the androgen receptor were significantly higher expressed in the tumors lacking the *HMGA2*-*NCOR2* fusion (Fig. 5D–F). Gene set enrichment analysis revealed a significant enrichment of the Gene Ontology term “skeletal system development” among genes higher expressed in the control group.

### Keratin-negative giant cell-rich soft tissue tumors

**Fig. 6:**
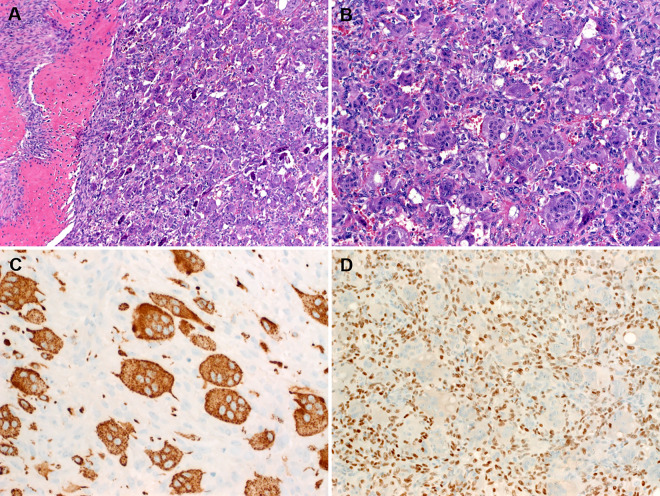
Representative histological images of conventional (keratin-negative, *HMGA2-NCOR2* fusion-negative) giant cell tumors of soft tissue. **A** A subset of cases contained a peripheral shell or islands of mature metaplastic bone. **B** Higher magnification of **A** (note close similarity to the case depicted in Fig. 3A). **C** CD68 highlighted the osteoclastic cells, but was less expressed in the mononuclear cells in this case. **D** Consistent nuclear immunoreactivity with SATB2 is seen in the majority of these tumors, note lack of expression in the osteoclastic giant cells.

The keratin-negative tumors (Tables 1 and 2) showed almost an equal sex distribution and affected patients with an age range of 11–78 years (mean, 46.7). Their sizes ranged from 1.5 to 4.5 cm (mean, 2.5). Histologically, they resembled GCT-B, and often showed a shell of mature bone (seen in 44.4% of cases) (Fig. 6A–C). In contrast to the keratin-positive cohort, these keratin-negative tumors lacked the peritumoral lymphoid reaction (*p* value = 0.01), frequently expressed SATB2 (3/4 cases; Fig. 6D) and lacked *HMGA2-NCOR2* fusions (0/7 cases). One case presenting as a minute dermal nodule revealed an *ALK-PPFIBP1* fusion and showed ALK immunoexpression (not shown). The H3.3 G34W antibody was negative in all cases. The main clinicopathological and molecular features of the two subgroups are compared in Table 3.

**Table 3:** Comparison of the main clinicopathological and molecular features of keratin-positive and keratin-negative giant cell-rich tumors of soft tissue.

Features	Keratin-positive tumors (*n* = 6)	Keratin-negative tumors (*n* = 9)	*p* value
Age range (mean)	14–60 years (34.7)	11–78 years (46.7)	0.48
Female: male (% females)	5:1 (83%)	4:5 (44.4%)	0.29
Size range cm (mean)	2–3 (2.5)	1.5–4.5 (2.5)	0.71
Mature bone formation (%)	0/6 (0%)	4/9 (44.4%)	0.10
Mitotic range (mean)	1–14 (7.2)	0–17 (4.1)	0.47
Peritumoral lymphoid reaction (%)	4/6 (66.7%)	0/9 (0%)	**0.01**
*HMGA2-NCOR2* fusion	6/6	0/7	**0.001**

Statistically significant *p*-values are in bold.

## Discussion

The nosologic classification of osteoclast-rich soft tissue neoplasms has been a matter of ongoing controversy and terminological evolution over the decades. This is due to the wide histogenetic spectrum of “osteoclastoma”-like lesions presenting within soft tissue, which encompasses metastatic giant cell-rich anaplastic carcinoma, giant cell-rich bone tumors extending into soft tissue (GCT-B, extra-osseous aneurysmal cysts, brown tumors, giant cell-rich osteosarcoma, and other rare variants), and osteoclast-rich variants of different specific soft tissue sarcoma types [4, 5].

Historically, the term “malignant giant cell tumor of soft parts” was used loosely to refer to a variety of soft tissue neoplasms, irrespective of the presence and the degree of cytological atypia and other features of malignancy [14]. However, due to the significant biological heterogeneity of the giant cell tumor category, these tumors underwent continuous refinement of diagnostic criteria [16]. When all the above entities and mimics are excluded, a subset of soft tissue GCTs cannot be otherwise classified into any specific sarcoma category. However, these definitionally bland-looking GCT-STs remain poorly characterized and the question whether they form a specific entity or merely represent a morphological pattern remains unsolved. The current WHO classification adopted for them the noncommitted term “giant cell tumor of soft tissue/GCT-ST” [2]. Older terminologies such as “malignant giant cell tumor of soft parts”, “giant cell tumor of low malignant potential” and “giant cell-type sarcomas” have been abandoned.

GCT-ST originates predominantly in the superficial soft tissues of the extremities followed by the trunk, head and neck, and other rare sites [16–18]. Adults in their fifth decade are predominantly affected with a wide age range (5–89 years) without sex predilection. Defining histological criteria of GCT-ST are multinodular superficial growth of uniform histiocytoid mononuclear cells lacking nuclear pleomorphism, admixed bland osteoclastic giant cells, variable hemosiderin deposits, and frequent metaplastic bone formation [2, 16–18]. A peripheral rim (shell) of woven bone is seen in almost half of the cases [2, 16–18]. Vascular invasion has been reported in up to 30% and aneurysmal features in a subset of cases [16–18]. Foamy macrophages may be observed in some cases. If strictly diagnosed, the biological behavior of GCT-ST is indolent with infrequent local recurrences (12%) and very rare or no metastases [2, 16]. The neoplastic mononuclear cells of GCT-ST display a similar immunophenotype as their osseous counterparts. They express monocyte-macrophage-associated antigen CD68, tartrate-resistant acid phosphatase, and smooth muscle actin, but not CD45, desmin, S100 protein, and lysozyme [16–20]. The osteoclastic giant cells express CD68, but not CD163 [28].

Although GCT-ST has traditionally been considered the soft tissue counterpart of GCT-B, based on the morphological similarities between the two entities, they lack the *H3F3* mutations, that characterize their osseous counterparts [6, 7, 19, 20] indicating different molecular pathogenesis. Further supporting this concept, studies have shown GCT-ST to share RANK and RUNX2 expression with GCT-B, but to show RANKL and SATB2 expression in only 25% of cases [20, 29]. The H3.3 G34W mutation-specific antibody, which has been developed as a valuable surrogate marker with high sensitivity and specificity for GCT-B, was negative in our cases, which practically rules out the *H3F3 H3.3 G34W* mutation (observed in 85–90% of GCT-B cases) [8, 10, 19, 20]. However, other *H3.3* mutations (seen in the remaining 10% of cases) are not excluded by negative H3.3 G34W immunostaining.

Keratin expression has been previously studied to only a limited degree in GCT-ST. Oliveira et al. reported variable keratin expression in 3 of 19 cases [17]. In the current study, keratin expression separated GCT-ST into two subgroups: one group (40% of all cases) displayed keratin expression and was uniformly positive for the *HMGA2-NCOR2* fusion, while the other subgroup lacked both features. Comparing the two subgroups by keratin expression, the keratin-positive tumors tend to present at a younger mean age (34.7 vs. 46.7 years), affect predominantly females (83% vs. 44.4%), lack mature bone formation (0% vs. 44.4%), display peritumoral lymphoid reaction (66.7% vs. 0%) and harbor the *HMGA2-NCOR2* fusion (100% vs. 0%), respectively. However, only peritumoral lymphoid reaction and the presence of *HMGA2-NCOR2* fusion appeared to be statistically significant due to the low number of cases. These distinctive demographic, clinicopathological and genetic features suggest two independent and separate tumor entities.

The high mobility group AT-hook 2 (*HMGA2*) encodes for a member of the non-histone transcriptional regulatory proteins involved in early developmental stages during embryogenesis [30]. Rearrangements involving *HMGA2* at chromosomal region 12q14.3 have been detected in a variety of benign mesenchymal and mixed tumors including lipomas and osteochondrolipomas, chondromas of soft tissue, uterine leiomyomas, salivary gland pleomorphic adenomas, and many other entities [31, 32].

*HMGA2* chromosomal breakages mainly involve the third long intron between exon 3 and exon 4 of the gene. The translocation frequently results in the formation of a truncated form of fusion transcript [31–33]. Loss of the C-terminal domain as a consequence of the chromosomal breakage is likely related to tumorigenesis. Although a plethora of fusion partners (*LPP, RAD51L1, NFIB, EBF1, PPAP2B, LHFP, NCOA2*, and others) have been reported [31–34], we are aware of only one recent case report of an *HMGA2* fusion involving *NCOR2* as a fusion partner (see below) [35].

The *NCOR2* gene, mapped to chromosomal region 12q24.31, encodes the nuclear receptor co-repressor 2 (NCOR2), a member of the thyroid hormone- and retinoic acid receptor-associated co-repressors family [36, 37]. As a member of a multisubunit complex, NCOR2 (AKA: silencing mediator for retinoid or thyroid-hormone receptors = SMRT and T_3_ receptor-associating cofactor 1 = TRAC-1) is involved in transcriptional silencing of certain target genes via chromatin remodeling [36, 37]. The putative chimeric HMGA2-NCOR2 fusion protein combines a DNA-binding motif encoded by *HMGA2* exons 1–3 in the N-terminal part with two repressor domains, a binding site for histone deacetylase 3 (HDAC3) as well as binding sites for multiple nuclear hormone receptors and transcription factors encoded by *NCOR2* exons 16–47 in the C-terminal part [36, 37]. Although the exact mechanisms through which the novel *HMGA2-NCOR2* fusion initiates tumorigenesis are unknown, a de-regulating effect on gene expression through interaction with other transcription factors is likely expected.

To the best of our knowledge, the novel *HMGA2-NCOR2* gene fusion has been reported only once before [35]. Brahmi et al. reported on a rapidly growing tenosynovial giant cell tumor of the hand of a 61-year-old female that responded to pexidartinib. Molecular profiling revealed *HMGA2-NCOR2* and *NCOR2-SUPT3H* fusions, but not the expected *COL6A3-CSF1* fusion [35]. The single histological image suggests a tenosynovial giant cell tumor and keratin expression is not mentioned in the case description [35]. Notably, we have never encountered this fusion among >800 soft tissue neoplasms we have analyzed routinely in our laboratory using the same TruSight RNA fusion panel since 2017, including >10 tenosynovial giant cell tumors (data not shown). This strongly suggests that this novel fusion represents a specific genetic marker defining this subset of keratin-positive GCT-ST. Although a comprehensive survey of other giant cell containing soft tissue and bone neoplasms is beyond the scope of the current study, it is remarkable that keratin expression appears to be a feature only of tumors harboring *HMGA2-NCOR2* fusions, among osteoclast-rich soft tissue tumors.

It is unclear what keratin expression in *HMGA2-NCOR2*-positive GCT-ST signifies. Certainly, they show no morphologic features to suggest epithelial origin, lack the cytologic atypia seen in osteoclast-rich carcinomas, and do not appear to arise in association with the epidermis or cutaneous adnexa. Furthermore, with the exception of keratins, these tumors were entirely negative for all other tested markers, corresponding to markers of endothelial, melanocytic, myogenic, myoepithelial, and neurogenic lines of differentiation. Similarly, the morphologic features of these lesions are wholly dissimilar from those of epithelioid sarcoma, and they show retained SMARCB1 expression. Finally, the distinctive genetic features of these keratin-positive tumors and absent expression of SATB2 would suggest that this does not represent simply aberrant keratin immunoreactivity in “garden variety” GCT-ST. Our cases share some clinical (predilection for young women) and immunohistochemical (keratin expression) features with the entity reported recently by Fritchie et al. as “xanthogranulomatous epithelial tumor”, but the two tumor types are morphologically very different [38].

Although limited by the panel-based approach, the expression profiling of our two cohorts showed significant differences, with a clear separation of the tumors on the basis of presence or absence of the *HMGA2*-*NCOR2* gene fusion. Genes significantly overexpressed in the tumors with *HMGA2*-*NCOR2* fusion included *TMPRSS2*, *CCND3* and *RUNX1*. Additionally, genes physiologically expressed in lymphocytes (e.g. *TCL1A*, *IRF4*, and *POU2AF1*) were significantly overexpressed, likely related to the lymphoid infiltrate seen in the *HMGA2-NCOR2* group. In contrast, the keratin-negative “control” group overexpressed *FGFR1* and *FGFR3* (among others) and were significantly enriched for the Gene Ontology term “skeletal formation”, findings consonant with the bone formation and SATB2 expression observed more frequently in this group. The one tumor showing *ALK* rearrangement clustered with the keratin-negative “control” group. Although no definite conclusions on the biological relevance of deregulated pathways can be drawn from this method, the pattern clearly shows differences on a larger scale level of gene expression comparing these two subgroups of tumors.

In summary, we have described a distinctive osteoclast-rich, keratin-positive tumor of the subcutaneous tissues, differing morphologically and immunohistochemically from conventional GCT-ST, and consistently harboring *HMGA2-NCOR2* fusions. We propose the descriptive term “*keratin-positive giant cell-rich soft tissue tumors with HMGA2-NCOR2 fusion*” for these unusual lesions. *HMGA2-NCOR2*-positive osteoclast-rich tumors should be carefully distinguished from other osteoclast-rich and keratin-positive superficial soft tissue tumors, in particular carcinoma, germ cell tumor metastasis and epithelioid sarcoma. The natural history of these rare tumors appears quite favorable, although additional study and longer clinical follow-up are needed.

## Supplementary information

Supplementary Table 1
