# Supplementary material for: Recurrent novel HMGA2-NCOR2 fusions characterize a subset of keratin-positive giant cell-rich soft tissue tumors
Source: Mod Pathol. 2021 Mar 19;34(8):1507–20. doi: 10.1038/s41379-021-00789-8 (PMC8295036; doi:10.1038/s41379-021-00789-8)
Supplement: Supplementary file 1 — Supplementary Table 1 [file 41379_2021_789_MOESM1_ESM.docx]

| **gene** | **log2 fold-change** | **adjusted p-value** |
| --- | --- | --- |
| **Genes higher expressed in *HMGA2*-*NCOR2* fusion-positive tumors** | | |
| *TMPRSS2* | 5,73883651 | 2,21E-14 |
| *TCL1A* | 4,56544026 | 0,00067663 |
| *IRF4* | 3,53436102 | 4,68E-10 |
| *NTF3* | 3,35506797 | 7,26E-08 |
| *POU2AF1* | 3,13778155 | 0,00196802 |
| *LCK* | 2,2921972 | 4,94E-09 |
| *MN1* | 2,20610191 | 0,00021703 |
| *ZBTB16* | 2,17495666 | 0,00083457 |
| *RCSD1* | 2,12911386 | 4,81E-14 |
| *IL21R* | 2,12266028 | 4,03E-08 |
| *ARHGAP20* | 2,02846946 | 5,87E-05 |
| *FCGR2B* | 1,95463175 | 2,18E-05 |
| *ST6GAL1* | 1,77121362 | 6,86E-07 |
| *RHOH* | 1,72421078 | 0,00763735 |
| *IKZF1* | 1,69071446 | 1,18E-14 |
| *ITK* | 1,64870201 | 2,06E-05 |
| *LYL1* | 1,53965892 | 0,0061071 |
| *FLT3* | 1,39093142 | 0,00066839 |
| *CIITA* | 1,32019775 | 2,73E-05 |
| *LRMP* | 1,31200421 | 1,51E-06 |
| *CBFA2T3* | 1,29659591 | 0,00218751 |
| *CMKLR1* | 1,28991137 | 5,76E-05 |
| *MYBL1* | 1,28599008 | 0,00043651 |
| *ARHGAP26* | 1,26878779 | 2,79E-19 |
| *ACE* | 1,21774111 | 1,16E-06 |
| *BCL3* | 1,20256924 | 0,00023573 |
| *CCDC88C* | 1,1638699 | 2,20E-08 |
| *INPP5D* | 1,1271951 | 3,40E-09 |
| *MYO1F* | 1,09762971 | 9,64E-08 |
| *KSR1* | 1,07470704 | 0,00068164 |
| *MAFB* | 1,05761788 | 0,00897996 |
| *GPR34* | 1,02503676 | 0,00216952 |
| *MAF* | 1,02463957 | 0,00244782 |
| *SYK* | 1,02289475 | 7,66E-13 |
| *HHEX* | 1,01495835 | 7,58E-09 |
| *HIST1H4I* | 0,97812748 | 0,00774643 |
| *FLT3LG* | 0,97143083 | 0,00105413 |
| *BCL2L1* | 0,92725096 | 2,13E-15 |
| *CCND3* | 0,87960853 | 3,70E-05 |
| *RUNX1* | 0,87376317 | 3,10E-06 |
| *ERG* | 0,86770804 | 0,00038328 |
| *MECOM* | 0,80290379 | 0,00373245 |
| *CD74* | 0,7971655 | 0,00972286 |
| *FLI1* | 0,76223337 | 1,51E-06 |
| *TACC3* | 0,76093 | 0,00087327 |
| *LMO2* | 0,75051857 | 0,00024925 |
| *CHST11* | 0,74130014 | 0,00732554 |
| *MYC* | 0,72292711 | 0,00039474 |
| *PML* | 0,70441126 | 0,00052684 |
| *SORT1* | 0,70132422 | 0,00255814 |
| *SEPT6* | 0,69220535 | 0,00014557 |
| *BCR* | 0,68061668 | 0,00189037 |
| *RNF213* | 0,66001199 | 7,72E-07 |
| *STAT6* | 0,58687762 | 0,00038152 |
| *TPM3* | 0,56465264 | 0,0064567 |
| *DUSP22* | 0,53917043 | 0,00099057 |
| *SEPT9* | 0,53652086 | 3,23E-05 |
| *ELF4* | 0,53304871 | 1,86E-06 |
| *C2CD2L* | 0,5326047 | 0,00100788 |
| *CBL* | 0,47078818 | 1,02E-06 |
| *MSN* | 0,33192178 | 0,00055278 |
| *MKL1* | 0,32226462 | 0,00476882 |
| **Genes higher expressed in *HMGA2*-*NCOR2* fusion-negative tumors** | | |
| *PCM1* | -0,27397037 | 0,00897996 |
| *AFF4* | -0,2936485 | 0,00052684 |
| *ZMYM2* | -0,33730299 | 0,00110356 |
| *ACACA* | -0,38538877 | 0,00266811 |
| *TP53BP1* | -0,39662555 | 0,00638096 |
| *BRAF* | -0,40686964 | 0,00040625 |
| *BCL9* | -0,41293853 | 0,00218751 |
| *TOP2B* | -0,41738452 | 5,76E-05 |
| *SIK3* | -0,42239501 | 0,00820611 |
| *PPFIBP1* | -0,48778145 | 0,00135575 |
| *YAP1* | -0,49617464 | 0,00774643 |
| *SEC31A* | -0,50676391 | 2,44E-07 |
| *BCAS3* | -0,52087084 | 0,00034128 |
| *KAT6B* | -0,55950742 | 9,92E-09 |
| *TEAD1* | -0,60835608 | 3,32E-07 |
| *BICC1* | -0,6518151 | 0,00984659 |
| *LRRC37B* | -0,65330937 | 0,00038197 |
| *FOXP1* | -0,6661089 | 0,00068164 |
| *COL1A1* | -0,70247843 | 0,00852214 |
| *CTNNB1* | -0,70259958 | 3,10E-06 |
| *TCF12* | -0,70638501 | 4,30E-07 |
| *CDH11* | -0,79589655 | 0,00269146 |
| *COL1A2* | -0,81428402 | 0,00052684 |
| *NFIB* | -0,82062928 | 0,00897996 |
| *WWTR1* | -0,84812719 | 4,41E-08 |
| *EML1* | -0,92834032 | 0,00161552 |
| *LHFP* | -0,95276155 | 0,0047326 |
| *MSI2* | -0,99722199 | 2,95E-07 |
| *SERPINF1* | -1,07778671 | 1,36E-05 |
| *MLLT3* | -1,09769006 | 0,00024925 |
| *AHI1* | -1,11777445 | 3,32E-07 |
| *AUTS2* | -1,14935762 | 2,74E-06 |
| *RUNX1T1* | -1,15942651 | 0,00030879 |
| *FRK* | -1,16963009 | 0,00774643 |
| *SETBP1* | -1,23372768 | 0,00042424 |
| *PBX1* | -1,25589349 | 0,00070323 |
| *SH3D19* | -1,42015028 | 1,10E-06 |
| *MLF1* | -1,44070963 | 1,07E-08 |
| *ANKRD28* | -1,4641575 | 8,73E-14 |
| *TRPS1* | -1,52163505 | 3,71E-12 |
| *TBX15* | -1,73771969 | 9,80E-09 |
| *PLAG1* | -1,75936543 | 0,00954362 |
| *FGFR1* | -1,83574034 | 6,68E-19 |
| *ID4* | -1,84385338 | 0,00052684 |
| *TET1* | -1,9771261 | 1,19E-24 |
| *LTBP1* | -2,03971826 | 1,99E-11 |
| *ZNF521* | -2,12154487 | 1,00E-15 |
| *BACH2* | -2,14162747 | 0,00068164 |
| *AR* | -2,14511968 | 0,00035915 |
| *ZFPM2* | -2,18378943 | 2,70E-05 |
| *HOXA11* | -2,35568982 | 0,00205814 |
| *HLF* | -2,45972736 | 0,00220885 |
| *NTRK1* | -2,85651574 | 4,32E-05 |
| *PRKG2* | -2,98202029 | 0,00066839 |
| *ELN* | -3,28619779 | 4,03E-08 |
| *OMD* | -3,45427675 | 1,06E-11 |
| *ETV1* | -3,51702039 | 2,07E-27 |
| *USP6* | -3,76881958 | 0,00034891 |
| *HAS2* | -3,88777532 | 1,08E-12 |
| *PAX3* | -4,44878933 | 0,00931912 |
| *HOXA13* | -4,56036319 | 4,28E-05 |
| *FGFR3* | -4,83868232 | 1,92E-19 |
| *HOXD11* | -6,64976215 | 1,51E-07 |
| *HOXD13* | -6,81304179 | 3,04E-06 |
